# Assessment of Dementia Risk and Health-Related Quality of Life in Patients Hospitalised in Geriatric Wards

**DOI:** 10.3390/jcm14217692

**Published:** 2025-10-29

**Authors:** Wiesław Fidecki, Irena Wrońska, Kornelia Kędziora-Kornatowska, Robert Ślusarz, Beata Dziedzic, Mariusz Wysokiński

**Affiliations:** 1 Laboratory of Clinical Skills, Medical University of Lublin, 20-081 Lublin, Poland; 2 Faculty of Health Sciences, Collegium Medicum Mazovian University in Płock, 09-402 Płock, Poland; i.wronska@mazowiecka.edu.pl; 3 Department of Geriatrics, Collegium Medicum in Bydgoszcz, Nicolaus Copernicus University in Toruń, 85-094 Bydgoszcz, Poland; kornelia.kornatowska@cm.umk.pl; 4 Department of Neurological and Neurosurgical Nursing, Collegium Medicum in Bydgoszcz, Nicolaus Copernicus University in Toruń, 85-821 Bydgoszcz, Poland; robert.slusarz@cm.umk.pl; 5 Department of Development of Nursing, Social and Medical Sciences, Medical University of Warsaw, 01-445 Warsaw, Poland; beata.dziedzic@wum.edu.pl; 6 Department of Fundamentals of Nursing, Medical University of Lublin, 20-081 Lublin, Poland; mariusz.wysokinski@umlub.edu.pl

**Keywords:** dementia, health-related quality of life, geriatric patient, geriatric ward

## Abstract

**Background:** Dementia is a common disease in the elderly, and its prevalence continues to increase worldwide. A significant proportion of patients with dementia are hospitalised due to comorbidities. Health-related quality of life (HRQoL) reflects overall health and is used in clinical trials, economic evaluations, and population health studies. The aim of this study was to assess the risk of dementia and quality of life related to the health status of patients hospitalised in geriatric wards. **Methods:** The study was conducted in geriatric wards of hospitals in the Lublin region. A total of 308 patients aged 65–98 years participated in the study. **Results:** The NOSGER scale evaluation of patients was at the average level of 75.82 points. The seniors showed best functioning in the area of disruptive behaviours (average 9.45 points), and the greatest deficits were found in the area of instrumental activities of everyday life (15.95 points). The cohort of patients assessed their overall quality of life at the average level of 3.16 ± 0.78 points and health status at 2.44 ± 0.77 points. The highest scores were given to the social domain (59.52 ± 13.69) and the environmental domain (56.96 ± 1.95). **Conclusions.** Psychophysical fitness was shown to decline in correlation with geriatric ward patients’ quality of life self-assessment.

## 1. Introduction

Ageing is the greatest risk factor for most chronic diseases, including cardiovascular disease, cancer, osteoporosis, arthritis, diabetes and neurodegenerative diseases such as dementia. Neurodegenerative diseases typically feature cellular and metabolic processes that accelerate in old age [1].

Dementia is a common disease in the elderly, and its prevalence continues to increase worldwide. A significant proportion of patients with dementia are hospitalised due to comorbidities. The proportion of patients with dementia ranges from 4 to 30% among patients discharged from general hospitals [2,3,4,5,6]. A multifactorial process that is always associated with cognitive decline and functional impairment, dementia has been found to be generally associated with higher hospital admission rates, longer hospital stays, and increased risk of mortality after admission, both in hospital and in home care [4]. As the disease progresses, people with dementia experience gradual dysfunction and loss of personal autonomy in addition to cognitive impairment. The diagnosis of dementia requires fulfilling criteria such as a loss of functional reserve and a decline in functional status, as well as memory impairment and/or other cognitive functions. Functional independence is an important component of quality of life from the perspective of the elderly. Showing functional decline, the elderly experience various negative effects, such as higher rates of hospital use, placement in care facilities and increased risk of death [3].

Health-related quality of life (HRQL) is an indicator of a person’s overall health and can be used in various contexts, such as clinical trials, economic evaluations of healthcare, or population health studies. There are many different concepts of HRQL; however, the one proposed by Patrick and Erikson is among the most frequently cited [5]. Patrick and Erikson defined HRQL as ‘the value attributed to life expectancy modified by impairments, functional states, perceptions and social capabilities affected by diseases, injuries or treatments’ [6]. Over the years, several models have been proposed to address this multidimensional concept [7,8,9], but it is the Wilson and Cleary model that is most widely used. The model comprises five domains, namely: biological variables, symptom status, functional status, overall health perception and overall quality of life. Moreover, it also takes into account individual and environmental characteristics. Each of the five domains has a direct impact on the next domain (i.e., biological variables influence symptom status, which in turn influences functional status, etc.), while environmental and individual factors influence all domains except biological variables [9]. Ferrans et al. proposed a modified version of said model, recognising that environmental and individual characteristics actually influence all five domains [7].

According to the model by Ferrans et al., health-related quality of life includes emotional well-being and social relationships in addition to physical condition. All these aspects may be significantly impaired in people with symptoms of dementia. Most recent theories account for the impact that environmental and individual factors exert on quality of life, hence the need to assess elderly people’s quality of life and its relationship with physical and mental functioning [10].

The study focuses on identifying the risk of dementia and assessing the quality of life in patients of geriatric wards. Early diagnosis of the elderly as well as the implementation of appropriate support and care measures are crucial from a public health perspective, as they aim to prevent the exacerbation of problems related to the functioning of senior citizens. These reports are important in terms of supporting vulnerable groups, such as the elderly, in order to improve their quality of life. However, it is the simultaneous use of the NOSGER scale, as well as assessment by means of the WHOQOL-Bref scale, that produces a comprehensive picture of the health and well-being of seniors. It also gives a better understanding of the relationship between seniors’ health and their perceived quality of life. This study fills a gap in Polish literature on the simultaneous assessment of dementia risk and the quality of life in older people, and its results are of practical importance for both hospital and community care, enabling the implementation of preventive measures and support for the physical, mental and social functioning of patients. This topic remains insufficiently researched in the Polish clinical environment, especially in the context of geriatric wards, where multiple chronic diseases and functional deficits often coexist.

The aim of this study was to assess the risk of dementia and quality of life related to the health status of patients hospitalised in geriatric wards.

The research hypothesis assumed that the greater the deficit in psychophysical functioning, the lower the subjective quality of life assessment in the study group.

## 2. Materials and Methods

### 2.1. Study Organisation and Participants

The study was conducted in geriatric wards of hospitals in the Lublin region (Poland). A total of 308 patients aged 65–98 years participated in the study. Inclusion criteria: age 65 and over, informed consent from the patient, and hospitalisation in a geriatric ward in the Lublin region. Exclusion criteria: age under 65, lack of consent from the patient, and hospitalisation in a ward other than a geriatric one.

It was a cross-sectional observational study.

### 2.2. Method

The research material was collected using the NOSGER tool (Nurses’ Observation Scale for Geriatric Patients). This questionnaire enables professional and non-professional senior caregivers to assess the physical, mental, and social condition of the patient quickly and easily. The scale consists of 30 questions and covers six dimensions: Memory; Instrumental Activities of Daily Living (IADL); Activities of Daily Living (ADL); Mood; Social Behaviour; and Disturbing Behaviour. Scale values are specified by numbers: from 1 (always) to 5 (never). A patient can receive a minimum of 30 points and a maximum of 150 points. The patient can gather between 5 and 25 points in each of the individual areas of the scale. The greater the number of points obtained in the observation, the worse the patient’s condition [11,12,13].

The reliability of the tool used in the research group of 308 elderly patients was checked by Cronbach’s alpha coefficient, which was 0.844 for NOSGER-total, 0.807 for ADL, 0.863 for IADL, 0.642 for Mood, 0.716 for Disturbing Behaviour, 0.870 for social behaviour and 0.956 for memory.

The second tool used in the study was the standardised WHOQOL-Bref questionnaire. The Polish version of the WHOQOL-Bref questionnaire developed by Wołowicka, Jaracz et al. was used in the study. This tool was designed for assessing quality of life in both healthy and sick people, for both research and clinical purposes. The questionnaire consists of 26 questions and facilitates obtaining a quality of life profile within the scope of four domains: physical, psychological, social and environmental. Two questions are analysed separately: question 1 referring to individual, general perception of one’s quality of life, and question 2 referring to individual perception of one’s health. The scoring system is positive—i.e., the more points, the higher the quality of life. Answers are given according to Likert’s 5-grade scale. Having been calculated according to the key, arithmetic means of scores obtained in the questionnaire range from 0 to 100 for each of the four domains and from 1–5 for the two questions: the former referring to general quality of life and the latter referring to health satisfaction [14].

### 2.3. Ethical Statement

The study was conducted in accordance with the requirements of the Helsinki Declaration.

The study was conducted in accordance with ethical principles. Participation in the study was voluntary and anonymous. Having read the information about the purpose and course of the study, a respondent could consent to participate. Nonetheless, if they changed their mind during the study and did not wish to continue, they could withdraw at any time without giving their reasons.

The study was conducted having obtained the consent of the Bioethics Committee at the Medical University of Lublin (Resolution No. KE-0254/45/02/2023).

### 2.4. Statistical Analysis

Statistical analysis and database searching were performed using Statistica 9.1 software (StatSoft, Poland). The results obtained in the analysis of quantitative variables are presented using the mean, median and standard deviation and in the analysis of qualitative variables using frequency and percentage. The normality of the distribution of variables was tested using the Shapiro–Wilk normality test. Differences between groups were assessed using the Mann–Whitney test for two groups, and in the case of three or more groups, the Kruskal–Wallis test was used; comparisons between individual groups were checked using the Mann–Whitney test with the Bonferroni correction. Pearson’s correlation coefficient was employed to determine the level of dependence between variables. In the statistical analysis, a significance level of *p* < 0.05 was adopted to determine the occurrence of statistically significant dependencies or differences.

## 3. Results

The characteristics of the study group are presented in Table 1. The study involved 308 patients, the majority of whom were women (63.97%).

The study analysed the correlation between the NOSGER scale and the WHOQOL-Bref scale (Table 2). A negative correlation was found between the scales in all component areas. Higher NOSGER scale values corresponded to lower WHOQOL-Bref scale values. These correlations were statistically significant, with the exception of the correlation between subjective health state assessment and three areas of the NOSGER scale: Disruptive behaviours, Social behaviour, and Memory. Although the vast majority of correlations were statistically significant, the effect size was mostly moderate or weak.

The strongest correlations (r > 0.5) show NOSGER total results to be moderately connected with the lower somatic sphere, psychological sphere and environmental sphere in patients’ quality of life. Activities of everyday life, instrumental activities of everyday life, mood and memory are moderately connected with the lower somatic sphere and psychological sphere in patients’ quality of life. Social behaviour is also moderately connected with the psychological sphere in patients’ quality of life. A strong correlation also occurs between social behaviour and the psychological sphere in patients’ quality of life.

The analysis of the study results is presented in Table 3. The NOSGER scale evaluation of patients was at the average level of 75.82 points. The seniors showed best functioning in the area of disruptive behaviours (average 9.45 points), and the greatest deficits were found in the area of instrumental activities of everyday life (15.95 points).

The analysis also included an assessment of patients according to selected sociodemographic variables (Table 4). When comparing the performance of the subjects by gender, it was found that men functioned slightly worse (average 77.41 points). Men were also rated worse in the component areas, with the exception of memory, where the results were similar (men 12.18 points vs. women 12.19 points). In none of the analysed areas was the difference statistically significant (*p* > 0.05).

When assessing the functional fitness of seniors using the NOSGER scale according to their age, people in the oldest age group were found to have the greatest functional fitness deficits (90.80 points). People aged 75–89 scored an average of 75.42 points. The best functional fitness was observed in people aged 65–74 (71.45 points). The statistical analysis showed a significant difference between the groups in all areas except Mood (*p* = 0.725).

The results were analysed according to the marital status of the patients. Widowed people (78.78 points) showed poorer functional fitness than married people (70.00 points). Widowed people also had lower functional fitness in each of the component areas. Based on the statistical analysis, it was shown that the difference between married and widowed people was significant in each area except for behavioural disorders (*p* = 0.140).

In the next stage of the study, the level of patients’ functioning was analysed according to their level of education. According to the NOSGER scale, the lowest level of functioning was found in patients with vocational education (82.50 points). On the other hand, seniors with secondary/higher education showed the best performance within the study group (61.36 points). When analysing patients’ scores in individual areas of the NOSGER scale, the greatest deficits were also found in people with vocational education. Based on the statistical analysis, a significant difference between the studied groups was found.

The last issue analysed was the determination of the level of functioning of patients depending on their place of residence.

The findings showed that the cohort of patients assessed their overall quality of life at an average level of 3.16 ± 0.78 points and their health status at 2.44 ± 0.77 points. The highest scores were given to the social domain (59.52 ± 13.69 points) and the environmental domain (56.96 ± 1.95 points). In the psychological domain, the score was 45.30 ± 13.54 points. The lowest scores were given to the somatic domain (37.85 ± 16.73). A detailed assessment of quality of life is presented in Table 5.

Table 6 presents the results of the WHOQOL-Bref questionnaire depending on the analysed variables. When analysing the quality of life depending on the gender of the respondents, it was found that men rated their health (2.48 points) and all four spheres of quality of life slightly higher than women. Women rated only their overall quality of life slightly better (3.16 points). However, this difference was statistically significant only in the environment domain (*p* = 0.030).

Age did not significantly differentiate the quality of life assessments of the surveyed seniors. However, the youngest age group (65–74 years) rated their quality of life the highest. The assessment of quality of life decreased with age. In the 90–96 age group, this assessment was at its lowest level. Older people who were married reported a better quality of life in all areas. In contrast, widowed people reported a lower quality of life. This difference was statistically significant in the psychological (*p* = 0.002) social (*p* = 0.000) and environmental (*p* = 0.010) domains.

The last issue analysed was the determination of the quality of life depending on patients’ level of education. Those with primary education were most satisfied with their health (2.48 points). In the assessment of quality of life and the four domains of the WHOQOL-Bref scale, the highest scores were obtained in the group of patients with secondary/higher education. The level of education significantly differentiated the quality of life of older people only in the somatic domain (*p* = 0.022).

## 4. Discussion

Conducting a comprehensive geriatric assessment, defined as a multidimensional, interdisciplinary diagnostic process, aims at determining the medical, psychological and functional capabilities of an elderly person in order to develop a coordinated and integrated treatment plan. This assessment is crucial for ensuring optimum care for elderly people in a hospital [15]. A geriatric ward is considered the most appropriate setting for providing this type of care [16]. It is believed that an age-appropriate environment, as well as medical staff with experience in caring for people with dementia, lead to better care outcomes, as reflected in a meta-analysis that found comprehensive geriatric assessment of patients in a specialist ward to increase the likelihood of returning home while reducing the number of admissions to inpatient care facilities or deaths and health deterioration [15].

Comprehensive geriatric assessment is specifically designed to identify and treat previously undetected geriatric syndromes that are known to carry a risk of adverse health outcomes. Cognitive impairment and dementia are recognised as the most prevalent geriatric syndromes and are often associated with many other syndromes and share common risk factors [17].

Dementia and cognitive impairment are global problems because, in older people, they often lead to functional disability and dependence. They place a burden not only on patients but also on their family carers, as well as on the healthcare system and economic resources. The problem of dementia is growing as populations are ageing. It is therefore crucial to recognise cognitive impairment at an early stage in order to implement pharmacological and non-pharmacological treatments that will slow down the process and prevent disability [18].

The problem of dementia in the elderly is the subject of many studies covering various aspects of this phenomenon [19,20,21,22,23,24,25,26].

With the projected increase in the proportion of elderly people in the Polish population, the incidence of neurodegenerative diseases, including dementia syndromes, will also increase. The onset of dementia symptoms quickly impairs the daily functioning of a patient, creating a need for assistance from others. In Poland, care for elderly people with dementia is mainly non-institutional and is provided by immediate family members in their own homes. Due to the declining care potential of Polish families, an increasing demand for formal forms of support in the care of elderly people with dementia is to be expected [27].

Assessment revealed deficits in functional performance among. However, the deficits were mainly manifested in physical functioning. To a lesser extent, there were deficits in dementia-related changes. This result indicates good mental performance in the group of seniors studied. The areas that received the highest scores were disruptive behaviour, activities of daily living and memory, all of which are the areas associated with the risk of dementia. Similar results were obtained in studies on the risk of dementia in other groups of seniors [28,29,30].

Functional fitness was comparable between men and women in our study. Studies conducted by Hebert et al., Kawas et al. and Edland et al. did not show a difference in the incidence of dementia in women and men [31,32,33]. However, in studies of patients in social care homes, the authors found that men scored slightly better on the NOSGER scale [34]. Similarly, in studies conducted in Sweden, the incidence of dementia was higher in women than in men, with the incidence of dementia differing after the age of 85 [35]. However, different results were obtained in studies of neurogeriatric patients, where women showed better functional performance [36].

Patients in the youngest age group (65–74 years old) were found to have the highest functional ability and were therefore the least likely to develop symptoms of dementia. The authors’ study showed that the functional ability of seniors deteriorates with age. The authors’ findings are consistent with those obtained by other researchers [37,38], which is confirmed by reports in the literature indicating an increase in the number of cases of dementia with age from approximately 1% after the age of 65 to approximately 40% after the age of 90. The incidence of dementia doubles approximately every five years [39,40].

Married people were found to function best, which is confirmed by a study conducted by Liu et al. [41] administered among American seniors. It was found that all groups of people who were not married, including those living in cohabitation, divorced/separated, widowed and never married, had a significantly higher probability of developing dementia during the study period than seniors in marital relationships. The study by Głowacka et al. also showed that marital status significantly differentiates functional ability, with married people being the least at risk of dementia [30]. Karakose et al. obtained different results in their study. The authors conducted an 18-year study in the United States and found that older people who remain married have a higher risk of dementia compared to seniors who have never been married, are divorced or widowed [42].

People with lower levels of education are more likely to show symptoms of dementia than those with higher levels of education [43,44,45]. The study showed that the level of education affected seniors’ psychophysical performance. Patients with secondary/higher education showed significantly better functional performance compared to those with primary or vocational education. Norwegian studies also showed that the increased risk of dementia associated with low education was differentiated by health and lifestyle factors assessed from early to late adulthood. Furthermore, 7–13% of the association between education and the risk of dementia in late life was mediated by health risk factors in early, middle and late adulthood and about 5% by lifestyle factors in middle and late adulthood. These studies also indicated a relatively stronger indirect effect of health risk factors and all mediators combined on the impact of low educational attainment on dementia risk among men compared to women [46].

Patients’ quality of life was reduced in geriatric wards. A study by Wróblewska et al. conducted in a group of patients in geriatric wards showed that the majority of respondents rated their quality of life as good (42.14%) [47]. The study by Jazayeri et al. showed that approximately 30% of older people had a high quality of life. The quality of life of more than one-third of older people was moderate, and approximately 34% of them had a low quality of life [48].

Studied women rated their quality of life lower than men. Similar conclusions were reached by Chen et al. in their study of 1278 elderly people in China [49]. In contrast, in the study by Machón et al. [5], a higher percentage of women rated their quality of life as good.

In general, the highest quality of life was reported by people in the youngest age group. Different results were obtained in the study by Kowalczyk et al. [50]. The authors showed that seniors aged 90+ rated their quality of life the highest.

Seniors with secondary or higher education were found to have a higher quality of life. Also, in a study conducted by Machón et al. [5], a higher percentage of seniors with secondary or higher education stated that their HRQL was good compared to those with lower levels of education. The study by Rao et al. also showed that the quality of life of elderly people increases with the level of education [51].

Older people’s quality of life was found to increase with higher levels of education. These results were also confirmed by Saurav et al. [52]. However, a different relationship between education and quality of life was found in the study by Kowalczyk et al. The authors found that as the level of education increases, the quality of life of older people decreases [50].

In the study conducted in India by Devraj Shilpa et al. on a group of seniors, it was shown that variables such as gender, level of education and marital status significantly affect the quality of life perceived by older people [53].

Elderly people’s functioning largely depends on their intellectual and mental abilities, which determine their independent performance in everyday activities and participation in social life. Chronic diseases, which are common in this age group, lead to a gradual decline in functional ability and quality of life. Hence, older people are more likely to be hospitalised, which often leads to a significant deterioration in their functional capacity. Periodic immobilisation and limited physical activity during hospitalisation contribute to a decrease in strength and physical capacity, which increases the risk of developing frailty syndrome. As a result, dependence on carers increases and quality of life further declines [54].

Compared to other European Union countries, Poland’s population is ageing faster, and poverty rates among older people, especially in rural areas, remain higher than in other European Union countries [55].

Therefore, the Polish socio-economic situation differs from that in some European Union countries, while education and marital status are linked to health and functional inequalities. People with better education are more likely to enjoy greater health capital and higher health awareness [56], which translates into a higher quality of life and a lower risk of developing chronic diseases, including dementia. In Poland, married people enjoy a higher level of well-being and better functioning, which is confirmed by population studies. On the other hand, single and widowed people are more likely to experience social isolation, depression and a poorer quality of life [57].

The findings indicate a need to implement comprehensive measures in geriatric wards aimed at maintaining patients’ functional fitness through early identification of the risk of dementia. Involving medical staff in patient mobilisation, individually tailored rehabilitation and psychosocial support can optimise the treatment of comorbidities, reduce the effects of hospitalisation and make a real contribution to reducing the risk of dementia and improving older people’s quality of life.

### 4.1. Implications for Clinical Practice

The findings allow to identify patients at high risk of dementia whose quality of life is reduced. Thus planning and implementing interventions is facilitated in order to improve seniors’ quality of life and optimise geriatric care and support. Health policy decisions regarding resource management in geriatric hospitals and in the community/home care system are therefore streamlined. It is also important to provide psychosocial support to patients and their families and to ensure interdisciplinary cooperation between medical staff, including doctors, nurses, psychologists and occupational therapists. The findings indicate a need to monitor and further assess patients’ functioning in geriatric wards in every dimension of their social context. They need to be regularly screened to implement preventive programmes and health education.

### 4.2. Limitations of the Study

However, the study had certain limitations. It was cross-sectional in nature, which does not allow for the assessment of changes in the level of dementia and quality of life over time and limits the possibility of drawing causal conclusions. In order to better understand the dynamics of these relationships, longitudinal studies are necessary to track changes in functioning and quality of life over time. The investigation covered only the Lublin region, and participants were recruited from specific geriatric wards, which limits the possibility of generalising the results to the entire population of Poland. In addition, the living conditions of seniors in the Lublin region, e.g., access to healthcare and the socio-economic situation of older people, may differ from those of other regions. The selection of specific socio-demographic variables resulting from the study design was another limitation. Further research is recommended, taking into account a wider range of factors such as health status, depression and social isolation. This may provide a more complete understanding of the challenges associated with ageing. It should also be remembered that the NOSGER scale is a preliminary assessment tool and, in case of doubt, more detailed diagnostic tests are necessary.

The study also had considerable strengths. It was innovative because it simultaneously assessed the risk of dementia (using the NOSGER scale) and the quality of life in patients hospitalised in Polish geriatric wards using the WHOQOL-BREF. This type of comprehensive assessment is rare in Polish literature and facilitates better understanding of the interrelationships between cognitive, physical and social functioning and elderly people’s quality of life. Furthermore, the assessment of dementia risk and quality of life in seniors is particularly important as it enables the identification of patients requiring early intervention, as well as the planning of measures aimed at improving the physical, mental and social well-being of seniors, which is important from a public health perspective.

## 5. Conclusions

Psychophysical fitness was shown to decline in correlation with patients’ quality of life in geriatric wards. The patients showed a moderate risk of developing dementia. The greatest deficits were found in physical fitness.

The findings highlight the importance of regularly assessing geriatric wards’ patients with respect to their psychophysical functioning and the need to implement preventive and therapeutic measures. It is vital to provide support for both patients and their families in order to improve seniors’ quality of life. Further research taking into account a wider range of factors affecting the functioning and well-being of older people seems necessary.

## Figures and Tables

**Table 1:** Sociodemographic analysis of the study group.

Variable		*n*	%	95% CI
Gender	Female	197	63.97	58.46–69.12
	Men	111	36.03	30.88–41.54
Age (years)	65–74	66	21.43	17.21–26.35
	75–89	217	70.45	65.13–75.27
	90–98	25	8.12	5.56–11.71
Marital status	Widowers/Widows	204	66.23	60.78–71.29
	Married	104	33.77	28.71–39.22
Education	Primary	241	78.25	73.31–82.49
	Vocational	42	13.66	10.25–17.92
	Secondary	21	6.82	4.50–10.20
	Higher	4	1.27	0.51–3.29

**Table 2:** Correlations between the NOSGER scale and the WHOQOL-Bref questionnaire.

WHOQOL-Bref Questionnaire	NOSGER Scale						
	NOSGER Total	Activities of Everyday Life	Instrumental Activities of Everyday Life	Mood	Disruptive Behaviors	Social Behavior	Memory
Subjective quality of life assessment	r = −0.497 ***	r = −0.480 ***	r = −0.501 ***	r = −0.494 ***	r = −0.271 ***	r = −0.429 ***	r = −0.342 ***
Subjective health state assessment	r = −0.156 **	r = −0.161 **	r = −0.138 *	r = −0.347 ***	r = 0.0174	r = −0.091	r = −0.098
Somatic sphere	r = −0.573 ***	r = −0.591 ***	r = −0.616 ***	r = −0.515 ***	r = −0.264 ***	r = −0.426 ***	r = −0.550 ***
Psychological sphere	r = −0.667 ***	r = −0.577 ***	r = −0.636 ***	r = −0.527 ***	r = −0.418 ***	r = −0.706 ***	r = −0.550 ***
Social sphere	r = −0.400 ***	r = −0.313 ***	r = −0.371 ***	r = −0.361 ***	r = −0.299 ***	r = −0.419 ***	r = −0.283 ***
Environmental sphere	r = −0.523 ***	r = −0.488 ***	r = −0.490 ***	r = −0.423 ***	r = −0.310 ***	r = −0.490 ***	r = −0.441 ***

r—Pearson’s correlation coefficient, * *p* < 0.05, ** *p* < 0.01, *** *p* < 0.001.

**Table 3:** NOSGER scale evaluation.

NOSGER Scale	M	SD	95% CI	Me	Min	Max
NOSGER total	75.82	23.51	73.18–78.46	74.00	30.00	134.00
Activities of everyday life	11.91	5.21	11.33–12.50	11.00	5.00	24.00
Instrumental activities of everyday life	15.95	5.47	15.34–16.57	16.00	5.00	25.00
Mood	12.13	3.54	11.74–12.53	12.00	5.00	22.00
Disruptive behaviors	9.45	3.45	9.07–9.85	9.00	5.00	25.00
Social behavior	14.16	5.22	13.58–14.75	13.00	5.00	25.00
Memory	12.19	4.39	11.70–12.69	12.00	5.00	25.00

M—mean; SD—standard deviation; 95% CI—95% confidence interval; Me—median; Min—minimum, Max—maximum.

**Table 4:** Sociodemographic variables and NOSGER scale evaluation (mean ± standard deviation).

Variables			NOSGER	Activities of Everyday Life	Instrumental Activities of Everyday Life	Mood	Disruptive Behaviors	Social Behavior	Memory
Gender	Female	M ± SD	74.92 ± 23.05	11.65 ± 5.20	15.71 ± 5.39	12.08 ± 3.43	9.29 ± 3.51	13.97 ± 4.96	12.19 ± 4.34
		95% CI	71.68–78.16	10.92–12.39	14.95–16.47	11.60–12.56	8.81–9.79	13.28–14.68	11.59–12.81
	Men	M ± SD	77.41 ± 24.34	12.37 ± 5.22	16.38 ± 5.60	12.25 ± 3.74	9.73 ± 3.34	14.49 ± 5.66	12.18 ± 4.51
		95% CI	72.84–81.99	11.40–13.36	15.33–17.44	11.52–12.93	9.11–10.37	13.43–15.56	11.34–13.04
	Statistical analysis		Z = −1.026 *p* = 0.304	Z = −1.204 *p* = 0.228	Z = −1.107 *p* = 0.268	Z = −0.418 *p* = 0.675	Z = −1.567 *p* = 0.116	Z = −0.742 *p* = 0.457	Z = −0.279 *p* = 0.779
Age	I. 65–74 years old	M ± SD	71.45 ± 23.00	10.59 ± 5.20	14.50 ± 5.52	12.09 ± 3.30	9.12 ± 3.24	13.77 ± 5.38	11.37 ± 3.91
		95% CI	65.80–77.11	9.31–11.87	13.14–15.86	11.28–12.90	8.32–9.92	12.45–15.10	10.42–12.34
	II. 75–89 years old	M ± SD	75.42 ± 22.91	11.90 ± 5.09	16.00 ± 5.32	12.09 ± 3.59	9.30 ± 3.28	13.94 ± 5.04	12.15 ± 4.40
		95% CI	72.36–78.49	11.23–12.59	15.30–16.72	11.62–12.58	8.87–9.75	13.27–14.62	11.56–12.74
	III. 90–96 years old	M ± SD	90.80 ± 25.02	15.48 ± 4.75	19.32 ± 5.27	12.56 ± 3.78	11.64 ± 4.67	17.08 ± 5.62	14.72 ± 4.79
		95% CI	80.47–101.13	13.52–17.44	17.14–21.50	11.00–14.12	9.71–13.57	14.76–19.40	12.74–16.70
	Statistical analysis		H = 11.359 *p* = 0.034 I < III **, II < III * ε^2^ = 0.031	H = 16.253 *p* = 0.0003 I < III ***, II < III ** ε^2^ = 0.047	H = 14.595 *p* = 0.007 I < III ***, II < III * ε^2^ = 0.041	H = 0.642 *p* = 0.725	H = 7.163 *p* = 0.027 I < III *, II < III * ε^2^ = 0.017	H = 7.481 *p* = 0.023 I < III *, II < III * ε^2^ = 0.018	H = 10.172 *p* = 0.006 I < III **, II < III * ε^2^ = 0.027
Marital status	Married	M ± SD	70.00 ± 22.68	10.80 ± 5.13	14.84 ± 5.57	11.44 ± 3.75	8.92 ± 2.85	12.72 ± 5.14	11.26 ± 4.24
		95% CI	65.60–74.42	9.81–11.81	13.76–15.93	10.71–12.17	8.37–9.48	11.72–13.72	10.44–12.09
	Widowed	M ± SD	78.78 ± 23.43	12.48 ± 5.17	16.51 ± 5.34	12.48 ± 3.38	9.73 ± 3.70	14.90 ± 5.12	12.66 ± 4.41
		95% CI	75.55–82.02	11.77–13.19	15.78–17.26	12.02–12.95	9.22–10.24	14.20–15.61	12.06–13.28
	Statistical analysis		Z = 3.081 *p* = 0.002, r = 0.176	Z = 2.742 *p* = 0.006, r = 0.156	Z = 2.453 *p* = 0.014, r = 0.140	Z = 2.334 *p* = 0.019, r = 0.133	Z = 1.473 *p* = 0.140	Z = 3.525 *p* = 0.000, r = 0.201	Z = 2.678 *p* = 0.007, r = 0.153
Education	I. Elementary	M ± SD	76.15 ± 23.02	11.93 ± 5.18	16.20 ± 5.36	12.12 ± 3.35	9.31 ± 3.44	14.19 ± 5.17	12.39 ± 4.37
		95% CI	73.24–79.08	11.28–12.60	15.52–16.88	11.69–12.55	8.87–9.75	13.54–14.85	11.84–12.94
	II. Vocational	M ± SD	82.50 ± 27.03	13.14 ± 5.78	16.61 ± 6.00	13.35 ± 4.19	10.90 ± 3.74	15.52 ± 5.84	12.95 ± 4.61
		95% CI	74.08–90.92	11.34–14.95	14.75–18.49	12.05–14.66	9.74–12.07	13.70–17.35	11.51–14.39
	III. Secondary/Higher	M ± SD	61.36 ± 15.11	9.64 ± 3.68	12.44 ± 4.41	10.20 ± 3.35	8.44 ± 2.21	11.60 ± 3.57	9.04 ± 2.89
		95% CI	55.12–67.60	8.12–11.16	10.62–14.26	8.82–11.58	7.52–9.36	10.13–13.07	7.85–10.23
	Statistical analysis		H = 12.404 *p* = 0.002 I > III **, II > III ** ε^2^ = 0.034	H = 6.133 *p* = 0.046 II > III * ε^2^ = 0.014	H = 11.571 *p* = 0.003 I > III **, II > III ** ε^2^ = 0.031	H = 10.839 *p* = 0.004 I > III *, II > III ** ε^2^ = 0.029	H = 12.352 *p* = 0.021 I < II **, II > III * ε^2^ = 0.034	H = 8.268 *p* = 0.016 II > III * ε^2^ = 0.021	H = 15.818 *p* = 0.0004 I > III ***, II > III *** ε^2^ = 0.045

Z—Mann–Whitney test; H—Kruskal–Wallis test; M—mean; SD—standard deviation; 95% CI—95% confidence interval; *p*—*p* value, * *p* < 0.05, ** *p* < 0.01, *** *p* < 0.001; r—effect size; ε^2^—effect size (epsilon-squared).

**Table 5:** Assessment of patients using the WHOQOL-Bref questionnaire.

Quality of Life	M	SD	95% CI	Me	Min	Max
Subjective quality of life assessment	3.16	0.78	3.07–3.24	3.00	1.00	5.00
Subjective health state assessment	2.44	0.77	2.36–2.53	2.00	1.00	5.00
Somatic sphere	37.85	16.73	35.98–39.73	38.00	0.00	81.00
Psychological sphere	45.30	13.54	43.78–46.81	44.00	6.00	81.00
Social sphere	59.52	13.69	57.98–61.05	56.00	6.00	100.00
Environmental sphere	56.96	12.22	55.60–58.34	56.00	25.00	94.00

M—mean; SD—standard deviation; 95% CI—95% confidence interval; Me—median; Min—minimum; Max—maximum.

**Table 6:** Sociodemographic variables and WHOQOL-Bref questionnaire evaluation (mean ± standard deviation).

Variables			Subjective Quality of Life Assessment	Subjective Health State Assessment	Somatic Sphere	Psychological Sphere	Social Sphere	Environmental Sphere
Gender	Female	M ± SD	3.16 ± 0.80	2.42 ± 0.72	37.61 ± 15.74	45.13 ± 13.72	59.06 ± 14.30	55.81 ± 11.82
		95% CI	3.05–3.28	2.32–2.52	35.40–39.83	43.20–47.06	57.05–61.07	54.16–57.48
	Men	M ± SD	3.14 ± 0.76	2.48 ± 0.86	38.27 ± 18.41	45.58 ± 13.26	60.33 ± 12.56	59.00 ± 12.71
		95% CI	3.00–3.29	2.32–2.65	34.81–41.74	43.09–48.08	57.97–62.70	56.62–61.40
	Statistical analysis		Z = 0.371 *p* = 0.710	Z = −0.479 *p* = 0.631	Z = −0.193 *p* = 0.846	Z = −0.314 *p* = 0.753	Z = −0.854 *p* = 0.392	Z = −2.158 *p* = 0.030, r = 0.123
Age	65–74 years old	M ± SD	3.21 ± 0.85	2.31 ± 0.72	40.10 ± 18.34	45.72 ± 14.35	59.82 ± 13.28	57.62 ± 12.54
		95% CI	3.00–3.42	2.14–2.50	35.60–44.62	42.20–49.26	56.22–62.87	54.54–60.71
	75–89 years old	M ± SD	3.14 ± 0.76	2.45 ± 0.78	37.68 ± 16.35	45.78 ± 13.12	59.54 ± 13.53	57.01 ± 11.96
		95% CI	3.04–3.25	2.35–2.56	35.50–39.88	44.03–47.54	58.05–61.60	55.41–58.61
	90–96 years old	M ± SD	3.08 ± 0.81	2.68 ± 0.74	33.36 ± 15.04	39.88 ± 14.27	56.80 ± 17.48	54.84 ± 13.87
		95% CI	2.74–3.42	2.37–2.99	27.15–39.57	33.99–45.77	49.58–64.02	49.11–60.57
	Statistical analysis		H = 1.048 *p* = 0.592	H = 5.016 *p* = 0.081	H = 3.024 *p* = 0.220	H = 3.531 *p* = 0.171	H = 1.291 *p* = 0.524	H = 1.016 *p* = 0.615
Marital status	Married	M ± SD	3.26 ± 0.80	2.47 ± 0.90	39.29 ± 18.23	49.15 ± 12.86	65.30 ± 12.89	59.75 ± 12.53
		95% CI	3.11–3.43	2.30–2.65	35.75–42.84	46.65–51.66	62.80–67.81	57.31–62.19
	Widowed	M ± SD	3.09 ± 0.77	2.43 ± 0.70	37.11 ± 15.90	43.32 ± 13.48	56.56 ± 13.16	55.54 ± 11.84
		95% CI	2.99–3.21	2.33–2.53	34.92–39.31	41.47–45.19	54.75–58.39	53.91–57.18
	Statistical analysis		Z = −1.653 *p* = 0.098	Z = 0.254 *p* = 0.799	Z = −1.208 *p* = 0.226	Z = −3.702 *p* = 0.002, r = 0.211	Z = −5.373 *p* = 0.000, r = 0.306	Z = −2.572 *p* = 0.010, r = 0.147
Education	Elementary	M ± SD	3.1 ± 0.76	2.48 ± 0.78	38.00 ± 16.44	45.52 ± 13.81	59.68 ± 13.47	56.95 ± 12.00
		95% CI	3.07–3.27	2.38–2.58	36.37–40.55	43.77–47.28	57.97–61.39	55.43–58.48
	Vocational	M ± SD	3.00 ± 0.88	2.28 ± 0.74	31.83 ± 16.50	41.78 ± 12.80	56.52 ± 12.26	54.69 ± 13.97
		95% CI	2.72–3.28	2.05–2.52	26.69–36.98	37.80–45.78	51.77–61.28	50.34–59.05
	Secondary/Higher	M ± SD	3.28 ± 0.84	2.36 ± 0.75	42.12 ± 18.01	48.96 ± 10.93	63.00 ± 12.56	60.88 ± 10.59
		95% CI	2.93–3.63	2.05–2.67	34.68–49.56	44.45–53.47	57.81–68.19	56.51–65.25
	Statistical analysis		H = 2.190 *p* = 0.334	H = 2.659 *p* = 0.264	H = 7.564 *p* = 0.022, I > II * ε2 = 0.018	H = 4.760 *p* = 0.092	H = 4.111 *p* = 0.128	H = 4.791 *p* = 0.091

Z—Mann–Whitney test; H—Kruskal–Wallis test; M—mean; SD—standard deviation; 95% CI—95% confidence interval; *p*—*p* value, * *p* < 0.05; r—effect size; ε^2^—effect size (epsilon-squared).

## Data Availability

The data that support the findings of this study are available from the corresponding author upon reasonable request.
